# Interfacial Transition Zones in Concrete: A Critical Review of Characterization, Damage Evolution, and Mechanical Response

**DOI:** 10.3390/ma19214715

**Published:** 2026-11-05

**Authors:** Shanjing He, Xiang Zhou, Lu Shen, Fan Wang

**Affiliations:** 1 School of Civil Engineering, Jiangxi University of Science and Technology, Ganzhou 341000, China; 2 School of Low-Altitude Technology and Engineering, Gannan College of Science and Technology, Ganzhou 341000, China; 9320240017@gnust.edu.cn; 3 Ma’anshan Engineering Technology Research Center of Engineering Monitoring and Safety Monitoring, Ma’anshan 243031, China; 4 School of Mining Engineering, Jiangxi University of Science and Technology, Ganzhou 341000, China; 5 Ganzhou Key Laboratory of Intelligent Construction, Gannan College of Science and Technology, Ganzhou 341000, China; 6 School of Civil and Safety Engineering, Wanjiang University of Technology, Ma’anshan 243031, China; 7 School of Minerals Processing and Bioengineering, Central South University, Changsha 410083, China

**Keywords:** concrete, interfacial transition zone, characteristic parameters, damage evolution, crack evolution, fracture process zone, mesoscale model

## Abstract

Local properties of the interfacial transition zone (ITZ) are closely related to damage evolution in concrete, but improvements in interfacial microstructure do not always translate into better overall mechanical performance. This review examines ITZ formation, multiscale characterization, damage evolution, and mechanical response through experimental, numerical, and interface-modification studies. ITZ boundaries and thicknesses depend on characterization parameters and identification criteria; interface quality therefore requires assessment of local properties and their spatial distributions. ITZ effects vary with damage stage and target response. Following preferential interfacial cracking, crack paths, localization, and macroscopic response depend on relative fracture resistance, weak-zone distribution, and loading conditions. The damage-controlling location can consequently shift among the ITZ, mortar, and aggregate, helping explain inconsistent improvements after interface strengthening. Links between measured ITZ properties, post-peak softening, and fracture-energy dissipation still require joint validation against local damage and macroscopic response. Priorities include identifying conditions for damage transfer, determining whether early interfacial cracks continue to contribute, and testing predictions across conditions using local parameters. This synthesis supports evaluation of ITZ contributions, model-parameter selection, and interpretation of interface modification, providing a basis for concrete design and optimization.

## 1. Introduction

Concrete is a typical multiphase cement-based composite [1] whose macroscopic mechanical behavior is jointly influenced by the properties of aggregates, cement paste, and their interfaces. Heterogeneity in constituent properties and spatial distributions produces different local tendencies for damage initiation and propagation; crack development and localization further affect load-bearing capacity and fracture behavior [2]. The interfacial transition zone (ITZ) forms between aggregate and cement paste. Its particle packing and pore structure differ from those of the bulk paste [3], as do its bonding and local mechanical properties [4]. Observations of local deformation and cracking near the interface relate these differences to strain concentration and crack-initiation sites [5]. However, the ITZ is not a uniform layer with fixed boundaries: its microstructure and mechanical properties vary with distance from the aggregate surface and local position, and different characterization methods do not identify identical extents [6]. Understanding its mechanical role therefore requires consideration of both initial local characteristics and damage evolution during loading.

Research on these relationships has progressed from initial ITZ characterization to damage observation and simulation during loading. Studies of particle packing and hydration explain interfacial microstructure formation [3], while comparisons of aggregate size, water-to-cement ratio, and age demonstrate its dependence on material conditions [7]. Nanoindentation combined with scanning electron microscopy relates microstructure to local mechanical properties [8]; comparisons of porosity and microhardness show that different parameters identify different transition ranges [9]. During loading, experiments and simulations of modeled recycled aggregate concrete associate local deformation and crack development with constituent phases [10]. Experimentally constrained mesoscale models further examine how mortar and interface parameters affect the overall response [11]. These studies provide a basis for connecting initial characteristics, local damage, and overall mechanical behavior, but the quantities and scales represented by different tests and models must be considered when comparing their results.

Previous reviews have organized these advances by characterization method, material system, and modification technique. Chen et al. [6] reviewed ITZ identification, physical and mechanical properties, and influencing factors, providing a basis for comparing characterization results. Xu et al. [12] linked treatment mechanisms to performance changes through microstructure and nanomodification. Memon et al. [13] compared methods for improving the ITZ and adhered mortar in recycled aggregates and noted that some studies did not adequately distinguish their contributions. A review of lightweight aggregate concrete discussed interface properties and modification strategies in relation to absorption, hydration, and aggregate characteristics [14]. These reviews already address links between the ITZ and mechanical behavior. The present review builds on them by organizing the evidence around damage evolution: it examines when preferential cracking near the interface supports an explanation of subsequent crack development and overall response, and how this relationship changes with relative phase properties and loading conditions.

Two issues remain central to explaining concrete fracture from local ITZ characteristics. First, the ITZ extents identified by different methods have different physical meanings [6], while interface parameters in models are constrained by constitutive formulations and calibration procedures [11]. The correspondence between measured characteristics and model inputs therefore requires clarification. Second, early interfacial cracking, subsequent formation of a dominant crack, and changes in macroscopic performance are distinct responses; one cannot establish the others alone. Numerical studies show that relative strength changes redistribute damage between the interface and aggregate [15], but these relationships still require comparison with local observations in specific materials. It is therefore necessary to establish which responses are affected by ITZ property changes at which damage stages, particularly whether links to post-peak softening and fracture-energy dissipation are supported jointly by local damage evolution and macroscopic results.

This narrative review identified relevant literature through topic searches in Scopus and Web of Science and by tracing the reference lists of included studies. Searches continued through September 2026 and covered topics including the interfacial transition zone, concrete, damage, fracture, and mesoscale modeling. Literature selection focused on local ITZ properties, damage evolution, and macroscopic response, with particular attention to experimental and numerical studies that clearly described their material systems, parameter acquisition methods, loading conditions, and observed quantities. Relevant reviews and classical fracture theories provided context. Modification studies reporting mainly overall performance changes were used to discuss treatment effects and associated factors; their results were not used alone to establish the ITZ contribution. The review included 85 references published between 1976 and 2026. Given the differences in material systems, test methods, parameter definitions, and loading conditions, the findings were compared by damage stage and target response rather than pooled into effect-size estimates.

This review examines the links among ITZ microstructure, damage evolution, and concrete mechanical behavior. It considers how damage in the ITZ, mortar, and aggregate affects the response of interest at different loading stages, and how the phase or region playing the principal role changes with material and loading conditions. Section 2 reviews ITZ formation, multiscale characterization, and boundary identification to clarify the physical meanings of local parameters. Section 3 follows crack initiation, path selection, localization, and load redistribution to examine the links between local damage and macroscopic response. Section 4 compares parameter sources, relative phase properties, and validation results, discussing the evidence for interfacial contributions and its implications for material optimization and model-parameter selection. Section 5 summarizes the main findings and key unresolved questions. This analysis supports selection of interface indicators for target mechanical properties, interpretation of modification effects, and appropriate model inputs, thereby informing concrete design and optimization.

## 2. ITZ Formation and Multiscale Characterization

### 2.1. ITZ Formation Mechanisms and Multiscale Characteristic Parameters

Disruption of cement-particle packing by aggregate surfaces is an important cause of ITZ formation. The initial arrangement near the interface is relatively loose, with corresponding changes in porosity and particle-size distribution [3]. Microbleeding and water-film effects can further increase water availability in some interfacial regions [6]. As hydration products form and fill pores, initial porosity differences can diminish [3]. Image analysis by Diamond and Huang identified statistical exclusion of cement particles near the interface, yet the observed ITZ porosity was only slightly higher than that of the bulk paste [16]. Packing differences therefore do not necessarily persist as marked porosity differences after hydration. Local particle arrangement and hydration conditions also affect calcium hydroxide (CH) distribution and the formation and continuity of calcium silicate hydrate (C-S-H) [12]. The ITZ thus generally forms a region of gradual transition in pore structure, composition, and local mechanical properties.

The relative importance of these formation factors differs among interface systems. Aggregate roughness changes adjacent particle packing and pore distribution [17]; absorption and prewetting of lightweight aggregates affect water exchange and hydration [14]; and aggregate size, water-to-cement ratio, and age also alter microstructural distributions [7]. Yin et al. combined microstructural characterization, microhardness measurements, and splitting tests to examine ITZs at different positions [18], indicating that sampling location is another important condition for comparing interface properties. Studies of carbonated modeled recycled aggregates likewise show different transition ranges for porosity and microhardness [9]. Interface evaluation should therefore specify material and sampling conditions and distinguish the magnitude of a property change from its spatial extent.

ITZ characterization should refer to clearly defined physical quantities. Porosity, pore size, and connectivity describe pore structure; porosity distributions reflect the combined effects of particle packing and filling by hydration products [3]. Electron-probe line scans identify spatial transitions in chemical composition [19]. Local mechanical tests provide different information: nanoindentation compares indentation moduli in old and new ITZs and adjacent paste [20], direct tension and shear tests evaluate interface strength under the corresponding loading modes [4], and miniature bending combined with SEM–DIC relates local deformation to fracture response [5]. These quantities involve different loading modes and scales and are not interchangeable. ITZ thickness is the apparent extent identified using a particular parameter and boundary criterion.

Characterization results require further conversion before use in models. Microstructure mapping relies on empirical relations between image or pore information and mechanical properties [21]; inversely identified parameters are constrained by the constitutive model and calibration data [11]. Accordingly, this review distinguishes parameters obtained by direct measurement, empirical mapping, and response calibration. Their mechanical interpretation is discussed in Section 4.

### 2.2. ITZ Characterization Methods and Boundary Identification

Because the ITZ exhibits spatial gradients and heterogeneity, characterization methods probe different features and scales. Microscopic imaging mainly provides information on pores, particles, and interfacial morphology; elemental analysis reveals spatial changes in composition; and local mechanical tests characterize deformation or resistance within the test region. Table 1 summarizes common methods, their principal parameters, uses, and limitations.

The boundaries in Table 1 correspond to transitions in microstructure, chemical composition, or local mechanical properties. CT identification of thin ITZ layers depends on voxel size, phase contrast, and segmentation. DIC mainly records local deformation and cracking; its use to identify thickness or inversely determine modulus also requires the mechanical assumptions to be specified. For example, DIC-derived ITZ extents and elastic moduli depend on the interval over which displacement changes abruptly and the fitting rule for the elastic segment [33]. Fluorescence microscopy by Shen et al. further showed substantial variation in detectable pore area fraction at equal distances around the same aggregate; a locally porous region cannot represent the average condition around the entire interface [34]. Comparisons between methods must therefore also consider sampling extent and statistical treatment.

Applying different methods to the same specimens within one study helps separate material differences from methodological differences. Zhan et al. [9] evaluated ITZ extents around untreated and carbonated modeled recycled aggregates using BSE porosity gradients and microhardness distributions, as summarized in Table 2.

Across the four specimen groups, BSE porosity gradients indicated apparent ITZ widths of approximately 60 μm, whereas microhardness distributions gave mean widths of approximately 86–99 μm. The two parameters therefore identified different transition ranges. Similar widths before and after carbonation do not imply unchanged porosity or hardness values. Width and the magnitude of property changes should be evaluated together to assess improvement or deterioration of the interface.

Table 3 further compares the identification bases of apparent widths reported for different materials, characterization parameters, and study conditions.

Comparisons made within a study using consistent methods and criteria are generally more directly interpretable. Jia et al. [35] examined aggregate size, water-to-cement ratio, and age. Shen et al. [36] used BSE imaging and nanoindentation, and Bao et al. [37] used Ca/Si gradients; both reported narrower transition ranges after carbonation. By contrast, the modeled recycled aggregate comparisons of Zhan et al. [9] showed no marked width change. Differences in material state, measured quantity, and boundary definition can affect these results. They do not establish which treatment is more effective or justify a general claim that carbonation necessarily narrows the ITZ.

Boundary-identification studies of simulated microstructures likewise show that thickness depends on the criterion used [39]. ITZ width should therefore be reported with material conditions, characterization parameters, and boundary criteria, and interpreted together with parameter magnitudes and spatial variations. Apparent width alone does not directly provide the local fracture resistance required for subsequent damage analysis.

## 3. ITZ-Related Damage Evolution and Its Links to Macroscopic Response

### 3.1. The ITZ and Damage Initiation

**Figure 1:**
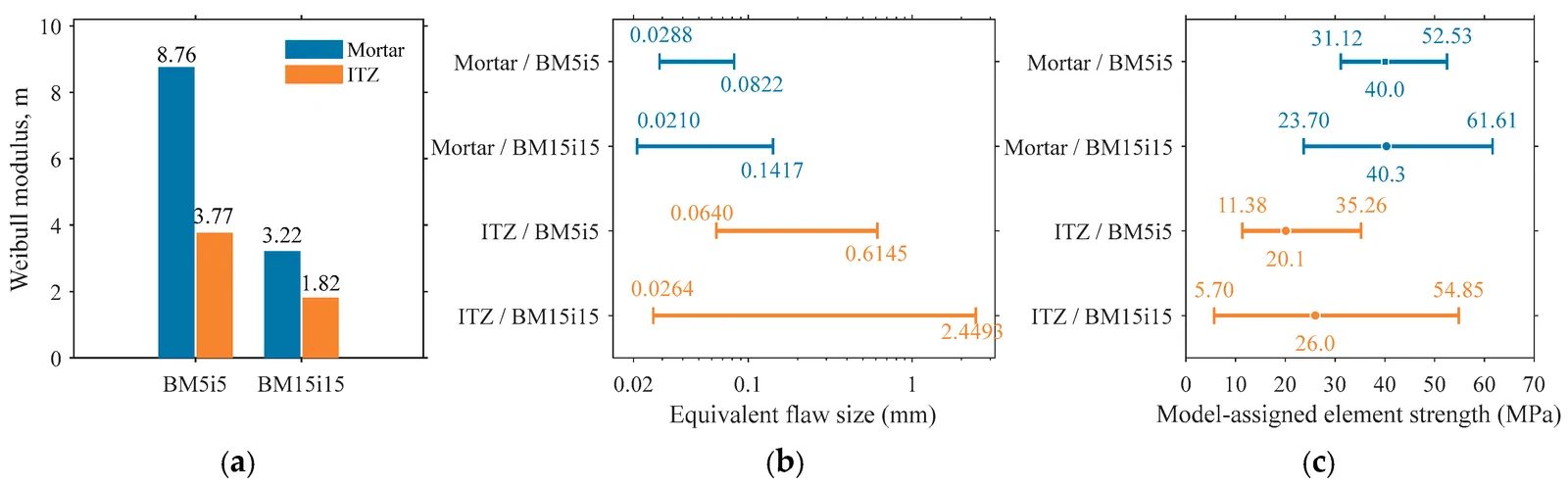
Comparison of stochastic mortar and ITZ parameters under two statistical settings: (**a**) Weibull modulus; (**b**) equivalent flaw-size range; and (**c**) upper and lower bounds and mean element strength. Replotted and reorganized from Table 2 of Bharadwaj et al. [40].

Preferential damage near the ITZ depends on local stress and the corresponding fracture resistance. Deformation incompatibility between aggregate and mortar can concentrate strain near their interface; microscale tests further associate high-strain regions with pores and locally less-hydrated regions [5]. In a stochastic three-phase mesoscale model, Bharadwaj et al. [40] assigned random strength distributions to mortar and ITZ elements and represented local weak regions through equivalent flaw sizes. Under both statistical settings, the ITZ had a lower Weibull modulus, a wider range of equivalent flaw sizes, and lower mean and minimum element strengths than the mortar (Figure 1). Figure 1 thus presents the assumed stochastic phase resistance used in the initiation analysis, rather than measured flaw statistics. The model relates equivalent flaw size to element strength through a Griffith-type relation [40]:

(1) $$\sigma =\frac{K_{c}}{\sqrt{\pi \bar{c}}}\varphi \left(\bar{c}/\omega \right)$$

where σ is the model-element strength, $K_{c}$ is the critical stress intensity factor, $\bar{c}$ is the equivalent flaw length, ω is a specimen geometric size parameter, and $\varphi (\bar{c}/\omega )$ is a geometric correction function. For the same $K_{c}$ and geometric correction factor, a larger equivalent flaw corresponds to lower local strength. In the loading simulations, ITZ elements were the first to become damaged under both statistical settings, illustrating how the prescribed differences in stochastic resistance affected initiation sites.

Observations of local deformation during loading provide experimental evidence of initiation sites. In modeled recycled aggregate concrete under uniaxial compression, DIC located the earliest bond cracks near the old and new ITZs [10], linking surface deformation to interfacial regions susceptible to cracking. Bimaterial three-point bending tests narrowed the observation to a single aggregate–mortar interface: SEM–DIC associated high maximum principal strains with pores and locally less-hydrated regions within the same field of view, where microcracks also initiated [5]. These observations demonstrate spatially heterogeneous initiation at the constituent-region and interfacial-microdefect scales. When interpreted with their respective loading modes, they provide complementary evidence for identifying vulnerable regions.

**Figure 2:**
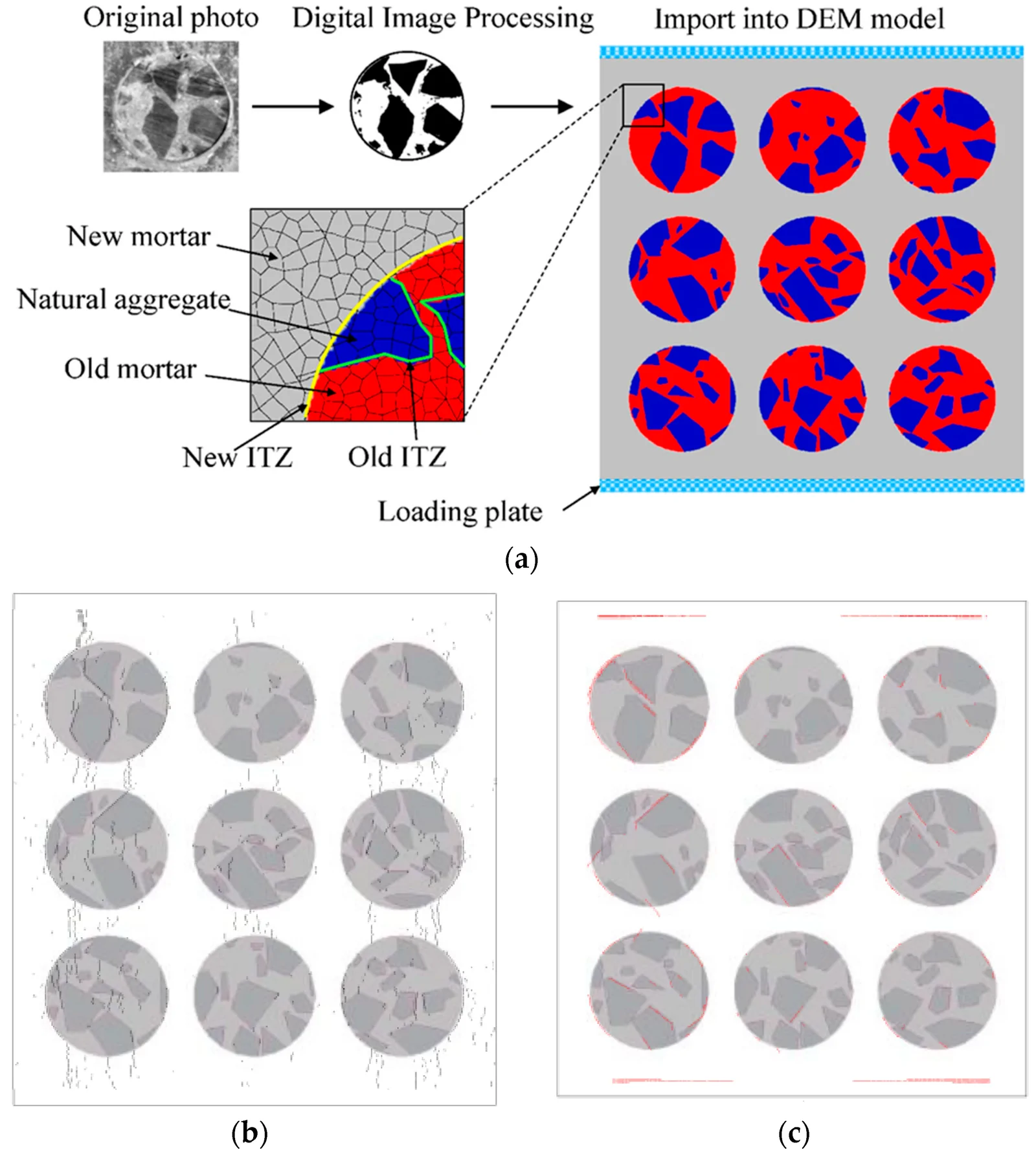
Mesoscale phase structure and simulated damage during early stable cracking in modeled recycled aggregate concrete: (**a**) phase structure reconstructed from specimen images; (**b**) tensile cracks at loading stage b; and (**c**) shear cracks at loading stage b. Adapted and reorganized from the relevant figures in [43].

Crack initiation in recycled aggregate concrete involves several candidate regions: old and new ITZs, adhered old mortar, and new mortar. Nanoindentation shows differences in local indentation modulus among the old ITZ, new ITZ, and adjacent paste [20]; variations in recycled aggregate source and interfacial microstructure also require interpretation within the specific material system [41,42]. For modeled recycled aggregate specimens, specimen structure and DIC results were combined to identify microcracks near the ITZ and in mortar during early stable cracking [43]. Mesoscale simulations in that study further distinguished failure modes: at the early stable-cracking stage (stage b), tensile cracks occurred more frequently in mortar, with some along the ITZ, whereas shear cracks were concentrated mainly along the ITZ (Figure 2). Under compression, local tension in interaggregate mortar can coexist with shear on inclined interfaces. The simulations therefore complement DIC crack-location data: both tensile and shear modes are needed to describe early damage allocation among phases, and counting only interfacial shear cracks underestimates the mortar contribution.

### 3.2. Crack-Path Selection, Coalescence, and Connectivity

**Figure 3:**
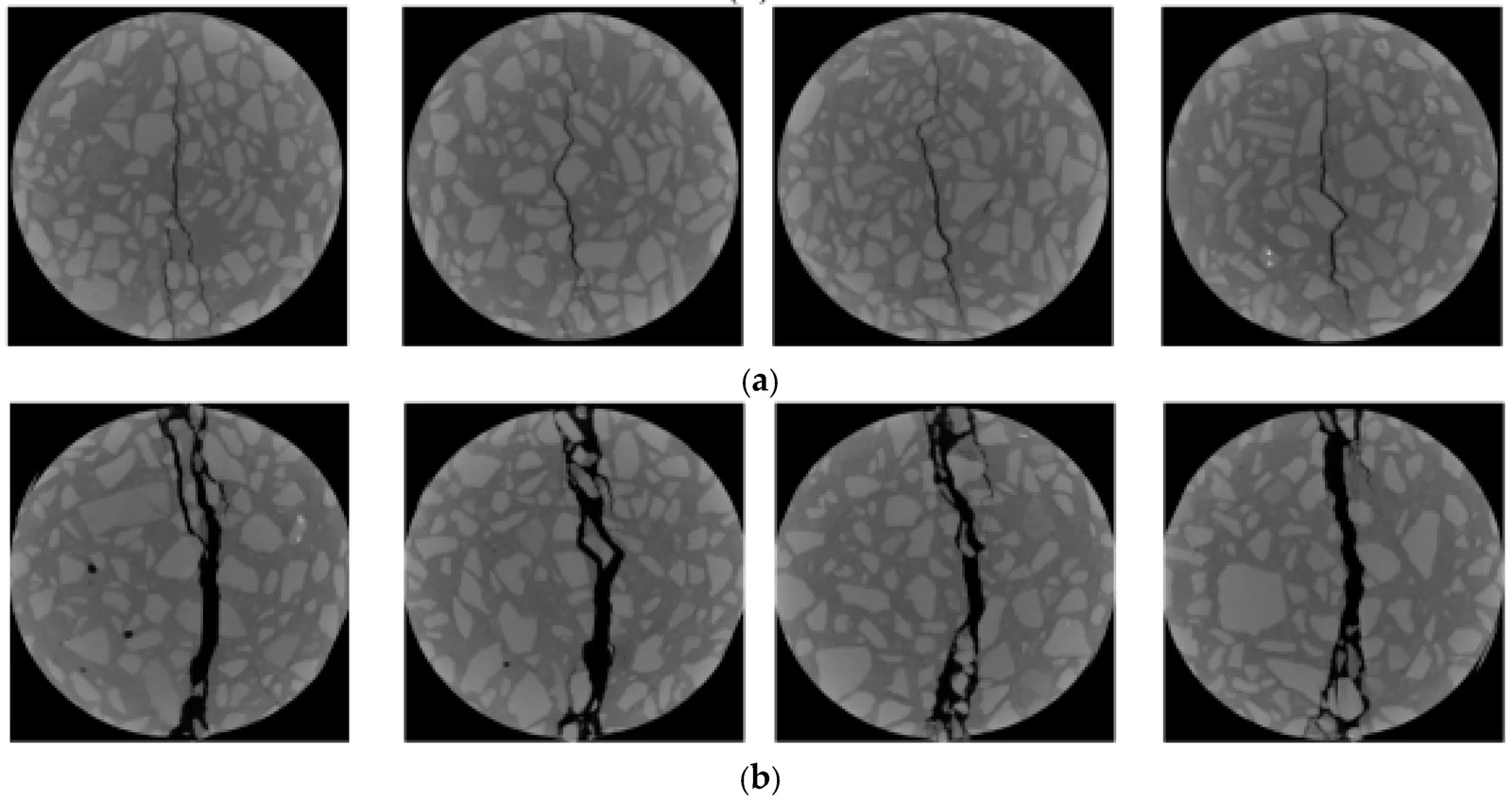
Post-splitting-failure CT sections of CA-group concrete of the same mix at different loading rates (water-to-cement ratio: 0.64): (**a**) 0.2 mm/min; (**b**) 2 mm/s. The images show the final spatial distribution of cracks, not the sequence of their initiation and propagation. Selected from Figure 10c,d of Zhang et al. [30].

Once initiated, cracks can propagate along the interface, enter mortar, or pass through aggregate. Comparisons of smooth river gravel and rough crushed stone associate aggregate-surface morphology and interfacial interlock with crack deflection, branching, and tortuosity [44]. Because lithology, aggregate strength, and surface characteristics also differ, these factors must be considered when interpreting path differences. Loading-rate comparisons provide another type of evidence. In Brazilian splitting tests on concrete of the same mix, cracks in the low-rate group occurred mainly along aggregate–mortar interfaces and in adjacent mortar. When the loading rate increased from 0.2 mm/min to 2 mm/s, post-failure CT sections showed more cracks crossing large aggregates and local crushing near small aggregates [30] (Figure 3). These images show final paths rather than the sequence of cracking, demonstrating different distributions of cracking among the interface, mortar, and aggregate as loading rate changed.

Interface-treatment studies examine how material changes affect local fracture. Carbonation treatment changed the bonding and fracture behavior of modeled recycled aggregate interfaces [45]. Changes in local interfacial resistance before and after treatment help explain whether cracks continue along the interface or enter adjacent material. In specimens containing multiple aggregates, this path selection also depends on aggregate geometry and loading conditions.

**Figure 4:**
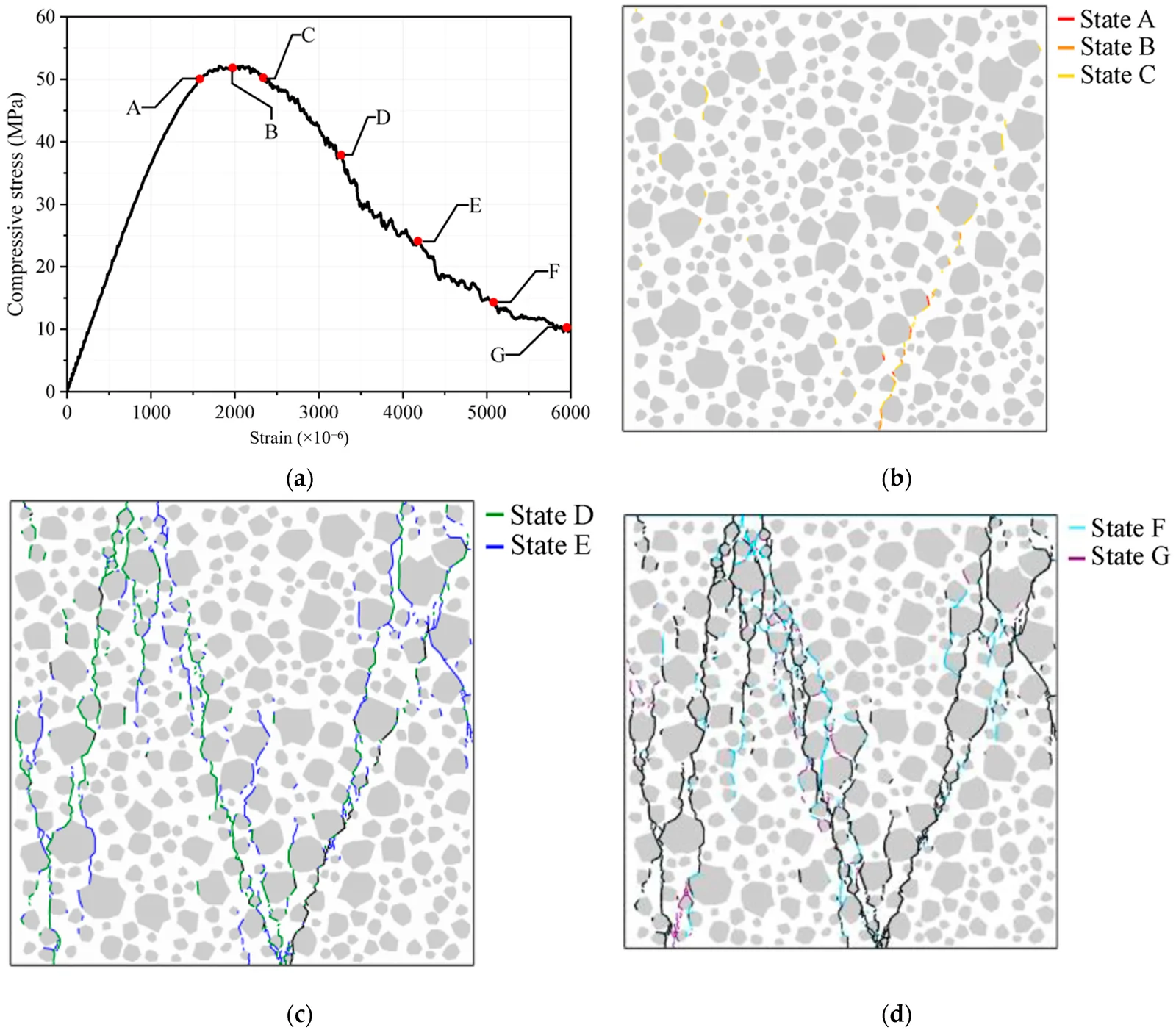
Evolution from distributed cracking to local coalescence in a mesoscale model under compression: (**a**) stress–strain curve with loading stages A–G; (**b**) crack distributions at stages A–C; (**c**) stages D–E; and (**d**) stages F–G. Adapted and reorganized from the relevant figures in [46].

Interfacial cracks extending toward adjacent weak regions must still cross intact connecting material. A smaller ITZ spacing can shorten mortar ligaments, but connection still depends on ligament resistance and local stress. Phase-field simulations in a microscale interface study predicted deflection, branching, and connection along aggregate boundaries [5]. The mesoscale compression simulations of Xiong and Xiao [46] linked crack distributions to loading stages: ITZ elements first failed at stage A, dispersed cracks formed at stages B–C, and subsequent failure of internal mortar interface elements promoted branching and coalescence, producing a main shear band by stage E (Figure 4). Preferential ITZ damage occurred even though the same cohesive parameters were assigned to the ITZ and internal mortar interfaces. Aggregate geometry and local stress can therefore affect the sequence of initiation and connection without a prescribed difference in cohesive resistance. The model also included post-cracking contact and friction, providing a basis for explaining continued load transfer during coalescence. These interpretations apply within the conditions represented by its discretization and calibrated parameters.

Geometric percolation, pore connectivity, and load-induced crack connectivity must be assessed separately. Hard-core–soft-shell models define geometric percolation by the formation of a spanning region of overlapping ITZ shells [47]. Its conditions depend on ITZ thickness, aggregate shape [48], particle-size distribution, and volume fraction [49]. Wood’s metal intrusion observations show preferential penetration into ITZ regions [50], but connected porous paste is not confined to the ITZ [51]. Internal-curing studies also show that pore connectivity and transport depend on material conditions [52]. Geometric connections therefore indicate potential crack-linking locations only. Actual coalescence still requires the connecting material to reach failure during loading and cannot be inferred directly from shell overlap or pore connectivity.

### 3.3. ITZ Damage Localization and Fracture Process Zone Evolution

As loading continues, some microcracks keep developing, and inelastic deformation and energy dissipation become concentrated within a limited region as a dominant crack forms and grows. Other early damage need not enter the main dissipative region. The fracture process zone (FPZ) must be identified from local deformation, damage, or energy-dissipation distributions. The fictitious crack model represents nonlinear fracture processes through a traction–opening relation across a crack [53], whereas the crack band model distributes softening and fracture-energy dissipation within a localization band of finite width [54]. Mesoscale lattice analysis further shows that dispersed interfacial damage does not fully coincide with the final concentrated dissipative region [55]. Applied to ITZ analysis, these findings emphasize tracking the subsequent opening of early interfacial cracks and damage in adjacent mortar, rather than inferring FPZ extent from initial crack counts.

**Figure 5:**
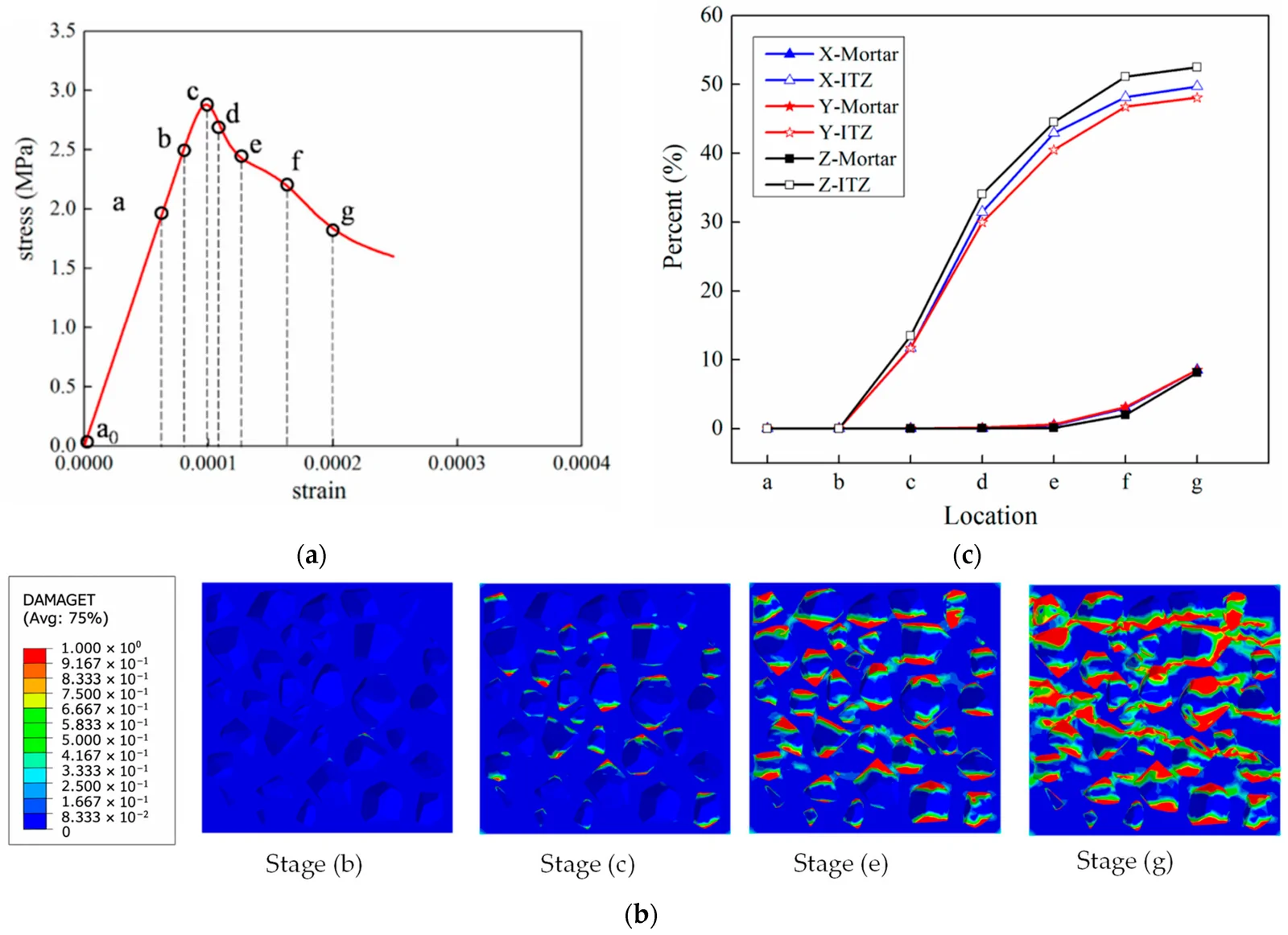
Damage localization in the ITZ and mortar under tension: (**a**) stress–strain curve with loading stages a–g; (**b**) spatial tensile-damage distributions at stages b, c, e, and g; and (**c**) proportions of damaged ITZ and mortar elements with loading stage under uniaxial tension along the x, y, and z axes. The original study classified elements with d_t_ > 0.9 as damaged; each proportion is normalized by the total number of elements in that phase. Adapted and reorganized from the relevant figures in [56].

Mesoscale tensile simulations show that expansion of the damaged ITZ region and formation of a localized crack band are related but distinct processes. Stage-resolved damage fields show progressive concentration from several near-interface regions into a crack band approximately perpendicular to the tensile loading direction [56]. Results for all three orthogonal loading directions indicate an earlier increase in the proportion of damaged ITZ elements, while the damaged mortar fraction rises mainly during softening (Figure 5). Spatial fields identify the localized region, whereas phase-wise curves quantify the fraction of each phase exceeding a prescribed damage threshold. Together, they show mortar participation in later localization. An intraphase damaged fraction is not an energy-dissipation share and cannot trace whether a particular early interfacial crack becomes part of the dominant crack.

Bimaterial three-point bending tests quantify local deformation near the interface at a smaller scale. The FPZ width identified from SEM–DIC relative-displacement profiles increased from approximately 25 μm at 25% of peak load to approximately 45 μm at peak load; relative displacement near the crack center also increased [5]. Expansion of the locally deforming region and continued opening of the central crack can therefore occur together, requiring both width and displacement distribution to be considered when interpreting localization. This width represents a local observation region in a bimaterial specimen. Comparisons with specimen-scale FPZs identified from damage or energy dissipation must distinguish the extents and criteria used.

After a dominant crack forms, damage and energy dissipation in the adjacent ITZ still vary with mesostructure. Two tensile models with identical material constitutive laws, but aggregate structures obtained from CT images and parametric generation, respectively, both formed a single dominant crack. Mortar energy dissipation was similar, whereas ITZ damage distributions and dissipation differed substantially [57]. Similar dominant-crack morphologies can therefore correspond to different energy allocations among phases, making phase-wise results necessary to evaluate the subsequent interfacial role.

Specimen size and aggregate structure also affect FPZ morphology. Classical size-effect theory describes changes in nominal strength with structural size [58]. Mesoscale simulations of notched specimens show that FPZ length along the propagation direction varies with specimen size, whereas transverse width is less size-sensitive [59]. Phase-field studies further examine size dependence in FPZ shape and extent [60], while aggregate content and size affect crack deflection and localization sites [61]. Quantitative FPZ comparisons therefore require loading mode, specimen size, and identification criteria to be specified, particularly when comparing local interface tests with specimens containing multiple aggregates.

### 3.4. From Local ITZ Damage to Macroscopic Mechanical Response

Local interfacial cracking changes load transfer between aggregate and surrounding paste. The overall response also depends on continued transfer through intact regions and crack faces. Lattice models represent post-cracking load bearing through crack-face bridging [62]. Three-dimensional lattice analysis further shows that the reduction in peak load and changes in ductility with increasing particle density depend on the assigned ITZ and matrix strengths; when their strengths are equal, this particle-density effect disappears in the model [63]. This comparison connects the overall effects of particle arrangement to relative phase strength. Another three-dimensional lattice study examined material properties and constituent proportions through simulations of compression, direct tension, and wedge-splitting tests [64]. In these models, the response after interfacial cracking depends on phase properties, remaining load-transfer paths, and loading mode.

**Figure 6:**
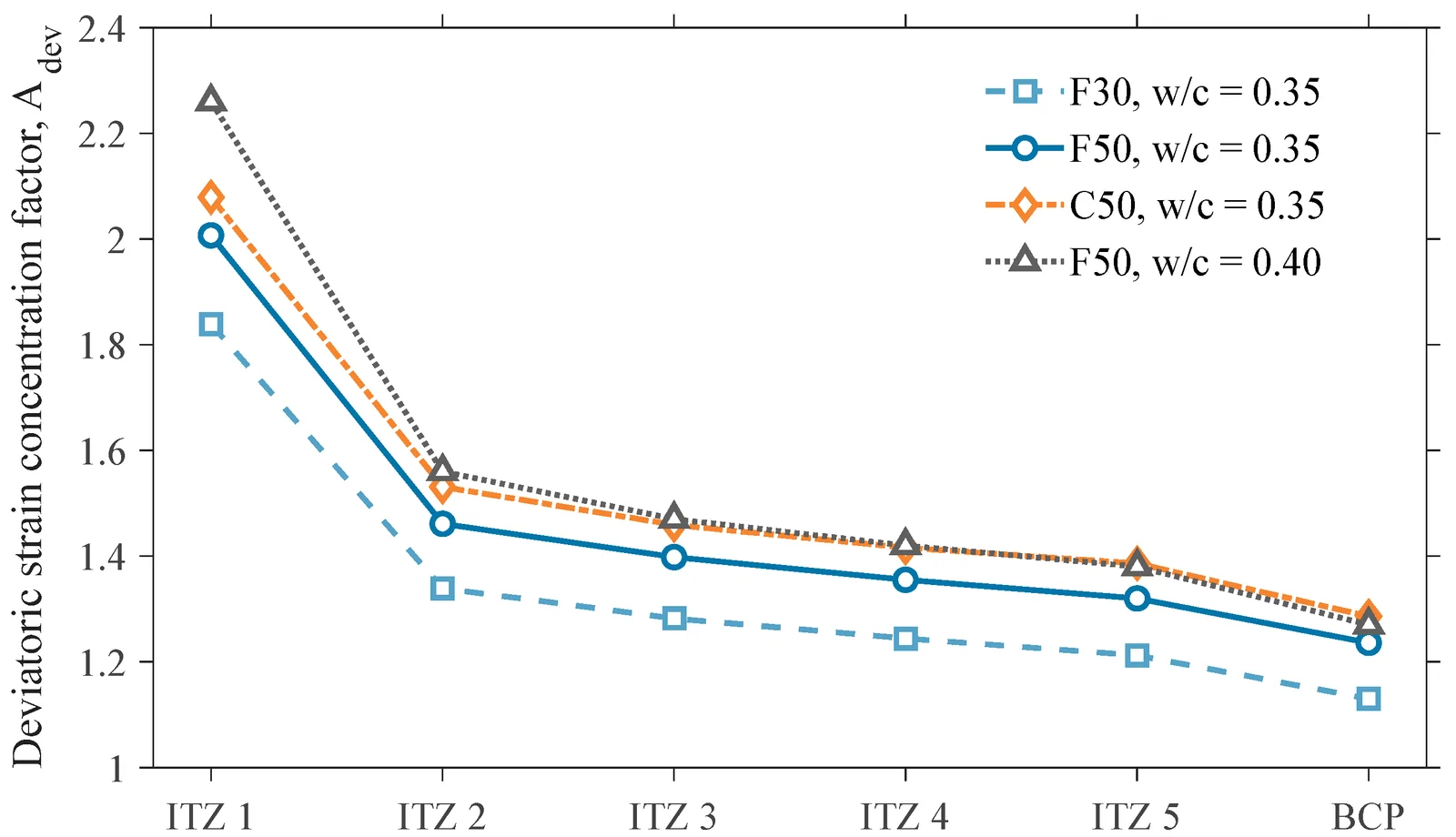
Layer-wise distribution of pre-damage deviatoric-strain concentration factors in a multilayer ITZ model and the effects of aggregate conditions. ITZ1 is the layer nearest the aggregate; BCP denotes bulk cement paste. Data reorganized from Tables 5, 7, and 8 of Gupta et al. [65]. Connecting lines only indicate trends across discrete layers.

The pre-damage distribution of deformation across interfacial layers provides the initial conditions for subsequent redistribution. In a multilayer ITZ model with a water-to-cement ratio of 0.40 and an aggregate volume fraction of 50%, the pre-damage deviatoric-strain concentration factor was 2.26 in the layer nearest the aggregate and 1.27 in the bulk cement paste. Factors in the other ITZ layers generally decreased with distance from the aggregate surface [65]. At a water-to-cement ratio of 0.35, F30 and F50 used the same particle-size distribution but aggregate volume fractions of 30% and 50%, respectively; F50 and C50 both had a 50% aggregate volume fraction but different particle-size distributions. Although the magnitudes varied, all settings retained higher pre-damage strain concentration near the aggregate (Figure 6). Subsequent damage analysis showed increased strain in the other ITZ layers and bulk paste after stiffness degradation of the near-aggregate layer [65]. This demonstrates redistribution of deformation as stiffness degrades. Load-sharing assessment additionally requires phase stresses; increased strain alone does not establish increased load.

**Figure 7:**
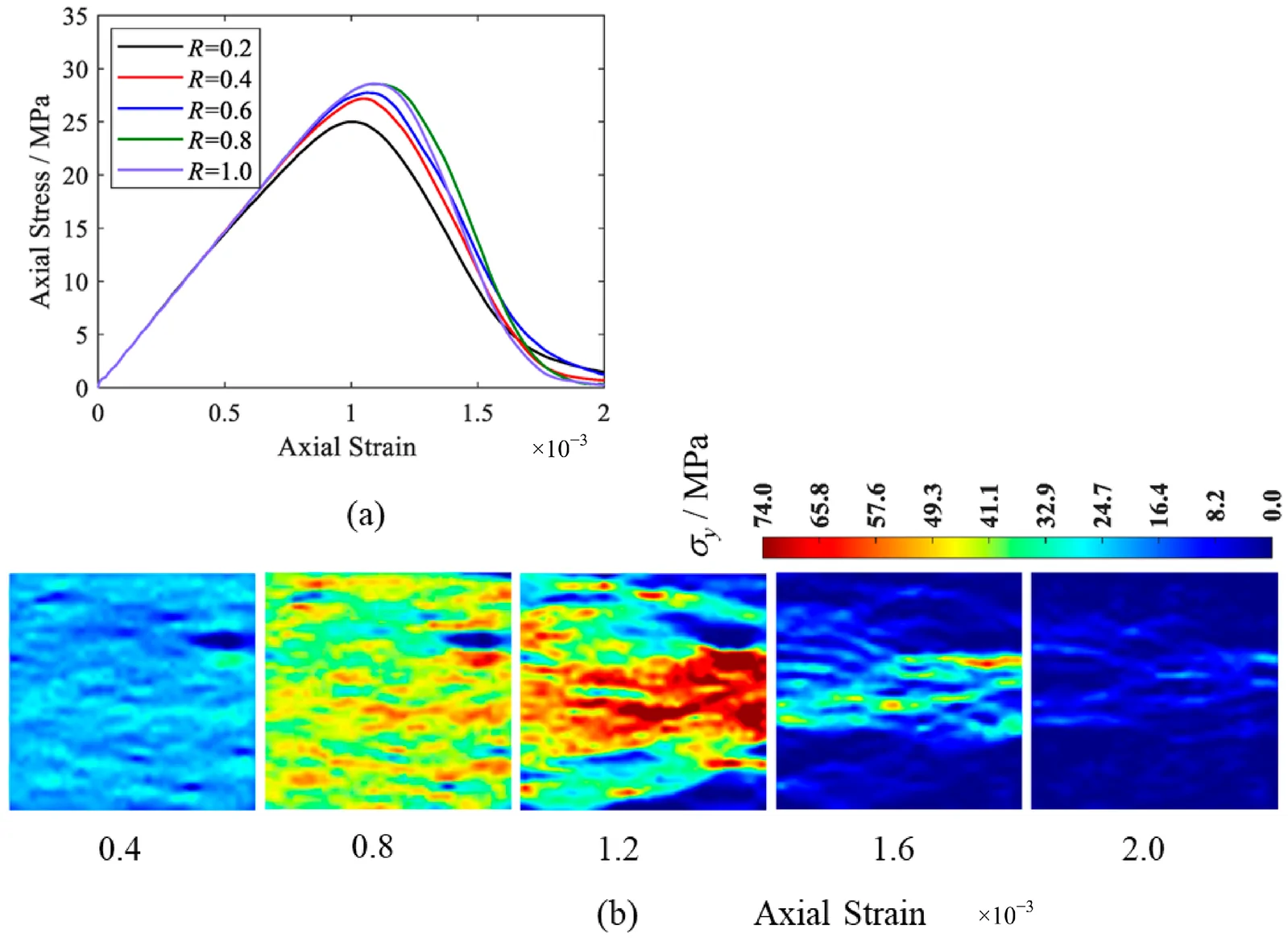
Compressive responses at different ITZ contact strengths and axial-stress-field evolution for a representative weak-ITZ setting: (**a**) axial stress–strain curves for different contact-strength reduction ratios, R; (**b**) axial stress fields at strains of (0.4, 0.8, 1.2, 1.6, and 2.0) × 10^−3^ for R = 0.2. Adapted and reorganized from the relevant figures in [66].

At the specimen scale, a two-dimensional discrete-element model compares overall responses and internal stress distributions at different ITZ contact-strength reduction ratios, R [66]. Stress–strain curves vary with R (Figure 7a). For R = 0.2, the high-stress regions and stress levels change as axial strain increases from 0.4 × 10^−3^ to 2.0 × 10^−3^ (Figure 7b). Stage-resolved crack records from the same study show early cracks mainly near interfaces, followed by increasing concentration in mortar at intermediate and later stages. The stress fields and crack records together describe internal stress changes accompanying damage development. Identifying local unloading requires damage and stress to be matched at the same stages to distinguish local degradation from the overall post-peak stress decline.

Subsequent damage in adjacent material also affects the overall response. Material-point simulations of uniaxial compression predicted damage around aggregates followed by increased mortar damage and associated some local failure with tensile stress [67]. A porosity-based discrete-element model showed that relative mechanical parameters of the ITZ and adjacent mortar changed the allocation of cracks between them [68]. Under these model conditions, mortar resistance and continuing damage form an important link between interfacial cracking and overall response. Evaluation of the subsequent ITZ role therefore requires phase-wise damage allocation to be related to peak load, post-peak softening, or fracture-energy dissipation.

## 4. Evaluation and Validation of ITZ Mechanical Contributions

### 4.1. Evidence for Different Mechanical Responses and Parameter Sources

Evaluation of ITZ contributions should distinguish observations from parameter comparisons. DIC records near-interface deformation and early cracking [10], whereas stochastic flaw models reveal how prescribed resistance affects initiation sites [40]. The former locates damage; the latter tests response changes under specified assumptions. Determining the role of a particular ITZ property requires comparison of initiation load, path, or subsequent response as that property changes.

Parameter sources determine what such comparisons can explain. Nominal tensile properties and fracture responses from bimaterial tests describe local fracture resistance under specified geometry and loading [69]; miniature bending tests further provide a basis for interfacial fracture-energy values [5]. These local quantities require suitable model representations before they can be used to analyze initiation and load bearing in concrete containing multiple aggregates. Gradient models convert microstructural grayscale or pore distributions into modulus and strength through empirical relations [21], whereas porosity-based discrete-element models require contact-parameter calibration [68]. Parameter changes in the former are constrained by the mapping relation; responses in the latter also depend on the contact law. If modulus and strength vary together according to a prescribed relation, the calculated response represents the combined parameter effect. Sensitivity to an individually varied parameter likewise needs to be related to its variation in actual materials.

The same interface change does not affect all mechanical quantities and responses equally. Wang et al. found that ITZ indentation modulus and hardness did not change in parallel after nanofiller modification: some groups had higher hardness than the bulk paste while their indentation modulus remained lower [70]. The choice of response measure is equally important when extending local-property comparisons to overall behavior. Phase-field models with an ITZ and models treating it as mortar both reproduced the compressive elastic segment and peak strength reasonably well, whereas incorporating locally constrained ITZ fracture properties improved post-peak simulations [5]. In a three-phase cohesive model, ITZ parameter changes substantially affected compression, while tension was relatively insensitive under the adopted mortar law and energy allocation [11]. Different response measures therefore require different interface information. Near-peak load-bearing comparisons cannot replace checks of post-peak softening and dissipation; loading mode and inelastic behavior in adjacent mortar must also be considered.

Table 4 distinguishes established relationships and evidence limitations by target response. Initiation observations identify vulnerable regions, controlled comparisons examine links between ITZ changes and response, and joint local and overall checks test whether an explanation extends to later stages. Where only early observations are available, conclusions should remain confined to supported stages; post-peak explanations additionally require continued crack opening, phase-wise damage, or energy dissipation.

### 4.2. Competition Among Phases and Shifts in the Damage-Controlling Location

Here, the “damage-controlling location” denotes the constituent phase or local region whose damage evolution plays the principal role in the mechanical response of interest at a given loading stage. Its identification requires continued crack opening, changes in phase-wise damage or dissipation, and their correspondence with the target response, rather than the earliest crack location alone. A change in the phase or region playing this role as material conditions or loading stages change constitutes a shift in the damage-controlling location.

“Competition among phases” describes selection among paths along the ITZ, into mortar, or through aggregate. This selection depends jointly on phase resistance, local loading, and geometry; it does not imply a universal competition coefficient. Descriptions of a “weak” or “strong” ITZ must also specify the reference phase and whether strength, stiffness, or fracture energy is meant. With mortar as the reference, tensile strength, elastic modulus, and mode-I fracture energy with consistent physical definitions and test modes can be expressed as:

(2) $$r_{t}^{i/m}=\frac{f_{t}^{i}}{f_{t}^{m}},\;r_{E}^{i/m}=\frac{E_{i}}{E_{m}},\;r_{G}^{i/m}=\frac{{G_{I}^{i}}_{c}}{{G_{I}^{m}}_{c}},\;\begin{array}{c} i\in \left\{I,a\right\} \end{array}$$

where I, m, and a denote the ITZ, mortar, and aggregate, respectively, and each denominator is the corresponding mortar parameter. Equation (2) defines the comparison variables, not a universal crack-transfer threshold. Strength and fracture energy require consistent loading modes and area definitions, while contact-model strength and stiffness must retain their constitutive meanings. Radially varying ITZ properties also require consideration of spatial distributions so that mean ratios do not conceal local weak regions.

Relative-parameter studies provide specific comparisons for interpreting different damage outcomes. A porosity-based discrete-element model compared ITZ and adjacent-mortar cracking at 0.9 times peak compressive stress; the relationship between their cracked fractions changed when the contact-parameter ratio increased from no greater than 0.68 to 0.8 [68]. This ratio involves both contact stiffness and strength and is not a universal tensile-strength threshold. A particle model varied relative ITZ and aggregate strengths separately and found different parameter dependencies for interfacial and transaggregate cracking [15]. Three-dimensional discrete-element simulations still produced aggregate damage at low ITZ strength; increasing ITZ strength increased mortar cracking but reduced aggregate cracking [72]. These findings do not require cracks to transfer to a fixed phase after interface strengthening. Instead, subsequent damage depends on adjacent-material resistance, local loading, and the failure modes allowed by the model. Table 5 retains the reported parameter ranges and loading conditions to facilitate comparison.

Phase-wise crack statistics can also affect identification of the controlling location. In models that record cracks through bond failure, the fraction of failed bonds within a phase should be distinguished from that phase’s share of all failed bonds:

(3) $$d_{i}(s)=\frac{N_{i}^{b}(s)}{N_{i}^{0}},\;p_{i}(s)=\frac{N_{i}^{b}(s)}{\sum_{j\in \left\{I,m,a\right\}}N_{j}^{b}(s)}$$

Here, $N_{i}^{b}(s)$ is the number of failed bonds assigned to phase i at loading stage s, and $N_{i}^{0}$ is the initial number of bonds in that phase. The total failed-bond count must be positive when calculating $p_{i}$, and bond categories must be mutually exclusive and consistently assigned. $d_{i}$ describes the extent of bond failure within the phase, whereas $p_{i}$ describes its share of all failed bonds. The two are not interchangeable: the interfacial share can decrease as mortar failures increase even if the interfacial failure count remains unchanged. Cross-study comparisons should therefore retain the original count-, length-, area-, or energy-based measure and loading stage, rather than referring to all measures as crack fractions. Equation (3) clarifies statistical differences; it neither equates bond counts with crack dimensions nor combines counts from different discretization scales. Identifying a shift in the damage-controlling location also requires continued crack opening and localization sites, supplemented by the target macroscopic response when overall performance is considered.

Aggregate structure and loading mode affect local stresses in addition to relative parameters. In concrete with constant paste content but different sand gradations, Rangaraju et al. did not generally observe the strength decline expected from greater interfacial extent and smaller particle spacing [75]. ITZ geometry alone therefore does not establish overall strength changes. Aggregate-type comparisons also involve lithology, morphology, and surface characteristics [44], while loading-rate tests show changing crack allocation between interfaces and aggregates [30]. Phase-parameter effects should thus be interpreted under the corresponding geometry and loading, with phase-wise damage and target response compared at matching stages.

Interface optimization under environmental actions requires consideration of different crack-driving mechanisms. Coupled hydro-mechanical peridynamic modeling shows that interface stiffness and fracture energy interact with the humidity field to influence branching and connection of drying-shrinkage cracks [76]. This provides a modeling basis for examining interface properties in shrinkage cracking and indicates that interface parameter combinations selected for external loading still require separate checks under changing humidity.

Interface-modification studies extend these parameter comparisons to practical material choices. Tests of modeled carbonated interfaces directly compare local fracture responses before and after treatment [45]. Acetic-acid and mechanical treatment of recycled aggregates changes adhered old mortar, absorption, and surface condition together; that study examined interfacial and overall properties [74]. The former helps identify changes in local resistance; the latter more closely represents the combined effects of a treatment process. Table 6 sets out treatment targets, measured results, and interpretive conditions to distinguish local interface improvement from the overall benefits of aggregate treatment.

For material optimization, these comparisons indicate that interface improvement should be assessed against the target performance. Reducing early-cracking risk calls for comparison of local interfacial resistance and specimen initiation load. Improving post-peak deformation capacity or fracture-energy dissipation additionally requires observation of continuing mortar and aggregate damage after interface strengthening. For recycled aggregate treatments, mix design should account for absorption changes, residual old mortar, and the effective water-to-binder ratio, rather than attributing differences caused by mixing conditions entirely to ITZ strengthening. If interfacial cracking decreases but the overall response improves little, possible explanations include increased damage in adjacent material, a reduced role of the original interface, or offsetting effects of other factors. Relative-parameter and phase-wise damage comparisons can therefore identify phases to prioritize for optimization, although treatment levels and parameter values must still be determined for the target material and loading conditions.

### 4.3. Joint Validation Against Local Damage and Macroscopic Response

Testing whether ITZ changes explain a target response requires separation of data used to determine parameters from data used to test predictions. Miniature bending tests supplied local fracture information for compressive phase-field analysis, which compared deformation fields and overall response [5], illustrating parameter transfer from local tests to specimen behavior. A microcantilever and XCT-based lattice study first calibrated the model against local load–displacement data and then predicted uniaxial tensile behavior [71]. The latter step demonstrates application under a different loading mode but does not establish validation against independent tensile experiments. These studies use different parameter bases; assessment should specify which quantities are directly measured, empirically estimated, or inversely determined, and which responses have not been used for fitting.

Parameter conversion first requires consistency in physical quantities and energy definitions. For a pure mode-I cohesive interface, fracture energy is the area under the normal traction–opening relation:

(4) $$G_{Ic}=\int_{0}^{\delta_{c}}T_{n}(\delta_{n})d\delta_{n}$$

Here, T_n_ is the normal traction, δ_n_ is the normal opening displacement, and δ_c_ is the displacement at complete loss of cohesive load-bearing capacity. Equation (4) distinguishes interfacial fracture energy from peak traction and the softening branch: similar fracture energies can correspond to different strengths and softening shapes, requiring checks against both initiation and post-peak data [11]. The initial stiffness of a zero-thickness interface has dimensions of stress per length and cannot be replaced directly by the elastic modulus of a finite-thickness layer. Conversion from volumetric dissipation to dissipation per unit crack area likewise requires an equivalent length. Tian and Chen used energy conservation to convert experimental ITZ mechanical parameters into cohesive-contact parameters for three-dimensional finite–discrete-element compression analysis [82], providing a specific example of such conversion. Grayscale-based gradient mapping [21] and hydration-based homogenization models [65] should distinguish measured, empirically mapped, and calibrated parameters and test conversion stability by varying the discretization scale, avoiding interpretation of numerical thickness effects as material ITZ changes. Zero-thickness interfaces, finite-thickness weakened layers, and gradient models define stiffness and dissipation differently. Even with local test inputs, phase-field and lattice models require the correspondence between parameters and discrete representations to be checked (Table 7).

Matching local characteristics and damage observations in space gives parameter checks a defined physical target. Correlative tomography offers a methodological example by registering information from different scales and modalities within the same volume [83]. In concrete, in situ three-dimensional loading observations have identified internal cracks and displacement distributions [84], while bimaterial tests provide local nominal mechanical properties [85]. Combining these results requires the observation region, interface geometry, and resolution to be specified. Parameters obtained from companion specimens also require matching material and preparation conditions. These checks allow the initial properties of a region to be related to whether its cracks continue growing, arrest, or are bypassed by the dominant crack.

Mishra et al. [73] combined in situ wedge-splitting tests, XCT, digital volume correlation (DVC), and phase-field analysis, providing an example that distinguishes model inputs, parameter calibration, and validation outputs. Model geometry came from measured mesostructure and boundary displacements from DVC. Some mortar parameters were measured, tensile and softening parameters were calibrated against bending response, and ITZ parameters were estimated from ratios to mortar properties and literature ranges.

For validation outputs, that study compared crack evolution, three-dimensional displacement fields, and crack opening displacement (COD). Measured boundary conditions improved agreement in crack morphology and COD, showing the importance of boundary conditions in explaining local prediction differences. However, the computational domain was an internal subvolume and did not directly provide the reaction force of the complete test. The case therefore supports joint checks of cracks and local deformation but cannot replace validation of the overall load response. A complete validation procedure would additionally compare overall responses not used in calibration.

Joint local and overall checks should compare continued crack opening, phase-wise damage or dissipation, and peak and post-peak responses at corresponding stages. The comparison in Section 3.3 between similar dominant-crack morphologies and different ITZ dissipation [57] shows how local results can distinguish explanations with similar overall predictions. If overall curves agree but damage allocation does not, parameter combinations, spatial structure, or constitutive representation should be examined.

Cross-condition prediction can further test these relationships. Model form and parameters are first established for a baseline condition. Unchanged phase parameters are then retained for the target condition, while inputs are updated using separately measured material properties, geometry, and loading conditions. The target response used for validation is excluded from parameter fitting; if the model is recalibrated, other data not used in calibration are needed for validation. Crack initiation, development, and macroscopic response are subsequently compared together, with discrepancies assessed against experimental scatter. Agreement at both local and overall levels strengthens the explanation for the specified response. If only initiation is reproduced, subsequent mortar or aggregate damage should be examined and the explanation restricted to the supported stage.

**Figure 8:**
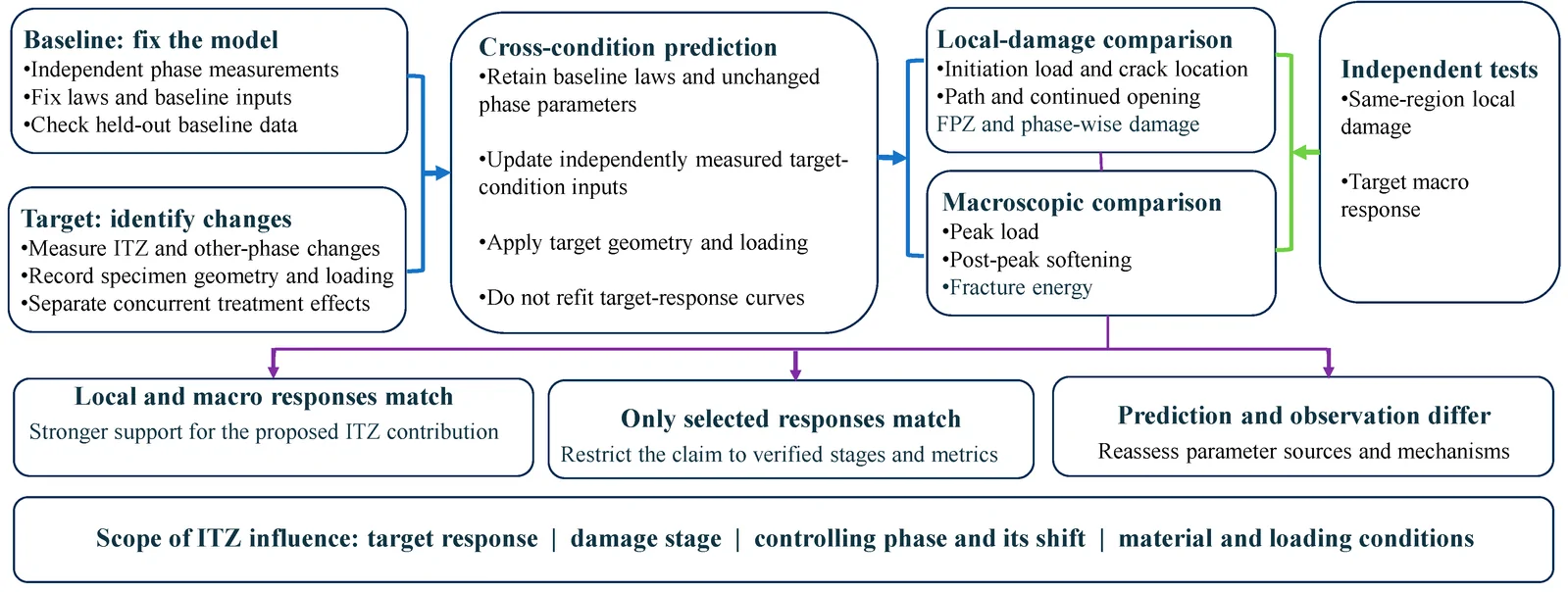
Proposed procedure for cross-condition prediction and joint validation of ITZ mechanical contributions, based on the literature analysis. The three lower branches represent alternative outcomes of the joint comparison, not successive stages. Blue arrows show model inputs and predictions, green arrows show independent observations, and purple arrows indicate comparison outcomes.

The interface treatments in Table 6 provide one class of cross-condition tests. If only ITZ properties change measurably after treatment, the corresponding inputs can be updated. Concurrent changes in old mortar, absorption, or effective water-to-binder ratio must also be quantified. Figure 8 accordingly proposes a validation procedure comprising baseline-model determination, target-input updating, and joint comparison of local and macroscopic responses. It also applies to predictions for unmodified concrete under different compositions or loading conditions.

## 5. Conclusions and Future Research Directions

### 5.1. Conclusions

(1) ITZ boundaries and thickness depend on characterization parameters, resolution, and identification criteria. Improved local properties do not necessarily imply a smaller apparent thickness; interface evaluation requires parameter magnitudes, spatial distributions, and the corresponding mechanical responses to be considered together.

(2) ITZ effects on damage are response-specific. Evidence of preferential near-interface initiation does not directly establish its role in subsequent paths, localization, or macroscopic response. Whether early interfacial damage continues to develop, and how adjacent mortar and aggregate participate, are central to linking different damage stages.

(3) Relative phase strength, stiffness, and fracture energy, together with weak-zone geometry and loading conditions, affect damage allocation. Interface strengthening can reduce interfacial cracking while changing stresses and damage in adjacent material. Its effectiveness should therefore be assessed against target mechanical performance, rather than interfacial crack reduction alone.

(4) Local tests and mesoscale models have established some links between ITZ properties and overall response. Measured, mapped, and calibrated parameters differ in physical meaning and constraints; assessment requires their sources, local damage, and macroscopic response to be checked together. Testing predictions under conditions not used for calibration is an important means of evaluating the reliability of these explanations.

### 5.2. Future Research Directions

(1) Conditions under which interface strengthening improves overall performance. Priority should be given to determining when changes in relative phase resistance and weak-zone spacing transfer continuing damage into mortar or aggregate, and how this affects post-peak deformation and dissipation. This would help identify which phases should be prioritized for optimization.

(2) Continued contribution of early ITZ cracks to later localization. Initial characteristics and crack opening should be tracked in the same region to distinguish continued growth, arrest, and bypass by the dominant crack. This would identify stages at which ITZ influence weakens and clarify its relationship with post-peak response.

(3) Cross-condition predictive capability of local ITZ parameters. Research should clarify conversion of experimental parameters into model inputs, quantify changes in other phases and loading conditions, and jointly test local damage and macroscopic responses not used in calibration, with emphasis on post-peak softening and fracture-energy dissipation.

## Figures and Tables

**Table 1:** Common ITZ characterization methods, measured quantities, and interpretive limitations.

Category	Methods	Principal Parameters	Main Uses	Limitations	References
Microstructural characterization	SEM and BSE image analysis	BSE-detectable pore area fraction; particle and hydration-product morphology; apparent transition extent	Compare pore enrichment and morphology with distance from the interface	Affected by preparation, resolution, thresholding, and section representativeness; cannot directly determine three-dimensional pore connectivity	[3,9,22]
Chemical composition	SEM–EDS and EPMA	Elemental distributions and ratios such as Ca/Si	Compare composition gradients; assist phase interpretation alongside other measurements	Affected by interaction volume and phase mixing; elemental ratios alone cannot establish phase content or local strength	[12,19]
Three-dimensional pore structure	X-ray CT and micro-CT	Resolvable three-dimensional pores and cracks, volume fractions, and connectivity	Analyze spatial morphology and connected paths of resolvable pores and cracks	Limited by voxel size, phase contrast, and segmentation; unresolved pores are excluded from statistics	[21,23,24]
Local mechanical characterization	Nanoindentation and microhardness tests	Indentation response, hardness, and modulus derived using a contact model	Compare local mechanical gradients and corresponding apparent transition extents	Affected by indentation scale, point spacing, and mixed phases; modulus or hardness cannot directly replace strength or fracture energy	[8,25,26,27,28]
Interfacial mechanical tests	Pull-out, splitting, shear, or microscale fracture tests	Nominal strength, fracture energy, or fracture resistance under specified specimen and loading conditions	Evaluate interfacial load bearing and fracture under a particular loading mode	Affected by interface geometry, loading mode, and stress-analysis assumptions; adjacent paste and aggregate effects are difficult to isolate	[4,5,29]
Damage-evolution observation	DIC, SEM–DIC, in situ loading, and ultrasonic tests	DIC: displacement, strain, and resolvable cracks; ultrasound: velocity, attenuation, or scattering	DIC tracks local deformation and cracking; ultrasound indirectly reflects internal changes	Local observations must be matched to initial structure; ultrasound alone cannot locate ITZ damage	[5,30,31,32]

**Table 2:** Apparent ITZ widths identified using different characterization parameters [9].

Modeled Recycled Aggregate Condition (Old-Paste Water-to-Cement Ratio in Parentheses)	Apparent Width from BSE Porosity Gradient (μm)	Apparent Width from Microhardness Distribution (μm)
RCA025 (0.25)	≈60	89 ± 19
Carb-RCA025 (0.25)	≈60	86 ± 14
RCA040 (0.40)	≈60	97 ± 21
Carb-RCA040 (0.40)	≈60	99 ± 15

**Table 3:** Apparent ITZ widths reported for different materials, characterization parameters, and study conditions.

Study	Material or Variable	Parameter and Method	Comparison or Conditions	Reported Apparent Width (μm)
Jia et al. [35]	Single-limestone-aggregate model concrete	BSE porosity and unhydrated-cement content distributions	Water-to-cement ratio: 0.35; age: 7 d; aggregate sizes: 5, 10, 20, and 30 mm	Approximately 25, 30–35, 45, and 50, respectively
Shen et al. [36]	Model cementitious interface	BSE and nanoindentation	Before/after carbonation	Before: 50–60; after: 20–30
Bao et al. [37]	Concrete	Spatial Ca/Si distribution by EDS	Before/after supercritical CO_2_ carbonation	Before: 47–79; after: 35–51
Xiao et al. [20]	Recycled aggregate concrete	Nanoindentation modulus distribution with microstructural observations	Old/new ITZ	Old ITZ: 40–50; new ITZ: 55–65
Momotaz et al. [38]	Rubberized concrete	Nanoindentation and SEM; EDS for supporting compositional analysis	Tire-rubber-particle–cement-paste interface; distinguish inclusion/exclusion of gaps and pores	ITZ: 50–65; weak region including gaps and pores: 80–100

**Table 4:** Links between the ITZ and different responses in representative studies, and limitations of the evidence.

Target Response	Representative Studies and Parameter Sources	Established Relationships	Evidence Limitations
Local fracture resistance and initiation threshold	Direct interface loading [4,29,69]; microscale fracture tests [5,71]	Measure local bond strength or fracture resistance for specified specimens and loading modes	Local resistance alone does not establish where concrete first becomes damaged; thresholds from different tests cannot be pooled directly
Initiation location	DIC and SEM–DIC observations [5,10]; statistical flaw model [40]	Observed near-ITZ deformation concentration and early cracking; modeled preferential interfacial damage	Location and sequence support ITZ involvement in initiation; pore, flaw, elastic-mismatch, and bonding effects remain to be distinguished
Crack path	Tests with different loading rates and aggregates [30,44]; relative-parameter models [68,72]	Propagation along interfaces or into mortar and aggregate varies with loading and phase conditions	Parameter comparisons establish sensitivity under specific settings, not a path-transfer criterion across material systems
Damage localization	Local FPZ measurements [5]; mesoscale models [55,56]; three-dimensional damage observations [73]	Record local deformation extent, phase-wise damage, or internal crack evolution, respectively	Few studies connect initial ITZ parameters, local resistance, and localization throughout loading in the same region
Overall peak response or strength	Microstructure mapping and parameter models [11,21]; interface and aggregate modification comparisons [26,74]	ITZ-related settings or treatments are associated with changes in overall mechanical measures	Mapped or inversely identified parameters are not independently measured resistance; treatments can also change old mortar, absorption, and mixing conditions
Post-peak softening and fracture-energy dissipation	Local tests and phase-field simulations [5]; multiscale and mesoscale models [57,65]	Some model links connect local interfacial fracture properties, phase-wise dissipation, and post-peak response	Local, phase-wise, and overall dissipation measures differ in scale; validation spanning measured ITZ changes and overall post-peak predictions remains limited

**Table 5:** Representative comparisons of relative ITZ parameters and damage responses.

Study and Condition	Parameters and Sources	Reported Comparison	Damage or Macroscopic Response	Conditions of Applicability
[68] Two-dimensional discrete-element compression	ITZ/mortar contact-parameter ratio from porosity mapping and calibration	No greater than 0.68 versus 0.8; phase-wise statistics at 0.9 times peak compressive stress	Relationship between ITZ and adjacent-mortar cracked fractions changes	Ratio involves contact stiffness and strength; sampled values cannot locate a universal threshold
[15] Particle-model compression	ITZ/mortar and aggregate/mortar strength ratios in a calibrated model	ITZ ratio: 0.52–1.38; aggregate ratio: 0.87–2.60	Stronger ITZ mainly reduces interfacial cracks; relative aggregate strength changes transaggregate failure	Scanned ranges are not ranges of control; response changes level off over different ranges
[72] Three-dimensional discrete-element compression	Relative ITZ parameters varied after experimental calibration; aggregate properties also examined	Relative ITZ parameter: 0.2, 0.4, 0.6, 0.8, 1.0, and 1.2	Aggregate failure can occur at low ITZ strength; stronger ITZ increases mortar cracking	Interpret parameters using the original contact law; account for aggregate strength and spatial structure
[21] Virtual tension in a gradient model	Measured microstructure mapped empirically to layered mechanical properties	Homogeneous/gradient representations with three or five layers; strength difference approximately 9.3% for five layers and below 2% for three	Property distributions affect predicted strength even with similar mean parameters	Layering and mapping assumptions affect results; not an independent prediction for measured modification groups

**Table 6:** Changes induced by interface modification and conditions for evaluating the ITZ contribution.

Material and Treatment	Main Targets and Concurrent Changes	Reported Local or Overall Results	Study Information and Interpretive Conditions	Reference
Recycled aggregate: acetic acid followed by mechanical rubbing	Adhered old mortar, absorption, and surface condition	ITZ microhardness, pores, and microstructure; concrete mechanical properties	Compare interfacial microhardness, pores, and overall performance; account for mortar removal and absorption changes	[74]
Recycled aggregate: acid, carbonation, acid–mechanical, acid–carbonation, and lime–carbonation	Old mortar and surface condition vary with treatment combination	Tension, compression, bending, and stress–strain comparisons	Assess overall treatment effects; local observations are needed to distinguish contributions from different regions	[77]
Recycled coarse and fine aggregates: thermal–mechanical treatment and accelerated carbonation	Residual mortar, absorption, and additional water	Physical and microstructural characterization; concrete performance	Combine physical and microstructural changes with performance; specify added water and water-to-cement ratio	[78]
High-strength recycled concrete: sodium-silicate immersion, cement–silica-fume slurry, and mechanical treatment	Absorption, abrasion, and surface condition; replacement level and size also examined	Aggregate properties and overall concrete performance	Compare treatment and aggregate-property improvements; interpret new-ITZ effects with surface and absorption changes	[79]
Recycled aggregate: nanosilica slurry	New ITZ and old-mortar surface; interfaces change differently	Nanoindentation, strength, and structural-scale strain	Local indentation and overall/structural responses are complementary; assess changes in each ITZ separately	[26]
Modeled recycled aggregate interface: carbonation	Old/new-mortar interface condition; new-mortar water-to-cement ratio	Flexural fracture response of the model interface	Supports comparison of local fracture before/after treatment; actual concrete also involves multiple interfaces and spatial structure	[45]
Natural aggregate: red-mud slurry	Wettability, surface pores, and interfacial bonding	Interfacial bonding, microstructure, and overall performance	Relates wettability and bonding to overall performance; findings apply to the natural aggregate and red-mud slurry tested	[80]
Recycled aggregate: mineral-admixture–magnesium potassium phosphate cement coating	Surface condition and interfacial microstructure; coating composition and treatment procedure also vary among groups	Coating and interface microstructure; concrete mechanical properties and durability	Compare coating, interface microstructure, and overall performance; account for composition and procedure differences	[81]

Note: This table compares different studies and does not rank treatment performance.

**Table 7:** Parameter definitions, conversion requirements, and validation responses for different ITZ model representations.

ITZ Representation	Parameter Sources and Studies	Conversion Checks	Responses Examined	Further Validation Priorities
Zero-thickness cohesive interface	Calibration and comparison constrained by tensile and compressive tests [11]	Initial stiffness relates traction to separation; define strength, fracture energy, and mixed-mode parameters using the cohesive law	Overall tensile/compressive responses and parameter sensitivity	Compare interface initiation, slip, and phase-wise damage in addition to overall curves
Finite-thickness multilayer ITZ	Layer properties from hydration and homogenization models [65]	Layer modulus is not zero-thickness interface stiffness; conversion from volumetric to areal dissipation requires layer thickness or equivalent length	Modeled layer-wise strain allocation and overall compression	Test predicted layer differences against local deformation and damage observations
Spatially graded ITZ	Micro-CT results mapped empirically to mechanical properties [21]	Retain spatial distribution, mapping relation, and resolution; do not replace gradients with one mean value	Virtual tensile responses under different gradients	Check correspondence of mapped properties with measured deformation and crack locations
Phase-field model constrained by local tests	Local fracture information from miniature bending and SEM–DIC [5]	Check experimental fracture energy against model dissipation definitions and specify length-scale effects	Local deformation, crack evolution, and overall compression simulations	Separate parameter-determination data from prediction-validation data
XCT-based lattice model	Microcantilever load–displacement calibration and XCT geometry [71]	Interpret element strength and dissipation using the constitutive law and discretization scale	Microcantilever calibration followed by uniaxial tensile prediction	Test cracking and load response using specimens or loading conditions excluded from calibration

## Data Availability

No new data were created or analyzed in this study. Data sharing is not applicable to this article.

## References

[1] Nilsen A.U., Monteiro P.J.M. (1993). Concrete: A three phase material. Cem. Concr. Res..

[2] Yılmaz O., Molinari J.-F. (2017). A mesoscale fracture model for concrete. Cem. Concr. Res..

[3] Scrivener K.L., Crumbie A.K., Laugesen P. (2004). The interfacial transition zone (ITZ) between cement paste and aggregate in concrete. Interface Sci..

[4] Jebli M., Jamin F., Malachanne E., Garcia-Diaz E., El Youssoufi M. (2018). Experimental characterization of mechanical properties of the cement-aggregate interface in concrete. Constr. Build. Mater..

[5] He J., Gao Z., Xiang Y., Chen J., Yang G., Xi B., Lei D., Zhao H. (2026). Microscale fracture analysis of concrete interfacial transition zone based on digital image correlation. Eng. Fract. Mech..

[6] Chen Q., Zhang J., Wang Z., Zhao T., Wang Z. (2024). A review of the interfacial transition zones in concrete: Identification, physical characteristics, and mechanical properties. Eng. Fract. Mech..

[7] Elsharief A., Cohen M.D., Olek J. (2003). Influence of aggregate size, water cement ratio and age on the microstructure of the interfacial transition zone. Cem. Concr. Res..

[8] Gao Y., Hu C., Zhang Y., Li Z., Pan J. (2018). Characterisation of the interfacial transition zone in mortars by nanoindentation and scanning electron microscope. Mag. Concr. Res..

[9] Zhan B.J., Xuan D.X., Poon C.S., Scrivener K.L. (2020). Characterization of interfacial transition zone in concrete prepared with carbonated modeled recycled concrete aggregates. Cem. Concr. Res..

[10] Li W., Xiao J., Sun Z., Shah S.P. (2012). Failure processes of modeled recycled aggregate concrete under uniaxial compression. Cem. Concr. Compos..

[11] Wang J., Jivkov A.P., Li Q., Engelberg D.L. (2020). Experimental and numerical investigation of mortar and ITZ parameters in meso-scale models of concrete. Theor. Appl. Fract. Mech..

[12] Xu Z., Bai Z., Wu J., Long H., Deng H., Chen Z., Yuan Y., Fan X. (2022). Microstructural characteristics and nano-modification of interfacial transition zone in concrete: A review. Nanotechnol. Rev..

[13] Memon S.A., Bekzhanova Z., Murzakarimova A. (2022). A Review of Improvement of Interfacial Transition Zone and Adherent Mortar in Recycled Concrete Aggregate. Buildings.

[14] Zhou J., Dong Y., Qiu T., Lv J., Guo P., Liu X. (2025). The Microstructure and Modification of the Interfacial Transition Zone in Lightweight Aggregate Concrete: A Review. Buildings.

[15] Zhao H., Wu Z., Liu A., Zhang L. (2022). Numerical insights into the effect of ITZ and aggregate strength on concrete properties. Theor. Appl. Fract. Mech..

[16] Diamond S., Huang J. (2001). The ITZ in concrete—A different view based on image analysis and SEM observations. Cem. Concr. Compos..

[17] Lyu K., Garboczi E., She W., Miao C. (2019). The effect of rough vs. smooth aggregate surfaces on the characteristics of the interfacial transition zone. Cem. Concr. Compos..

[18] Yin D., Zhang M., Xiong B., Zhang S. (2025). Study on the physical and mechanical properties of concrete interfacial transition zones (ITZ) with consideration of positional effects. Constr. Build. Mater..

[19] Tian Y., Tian Z., Jin N., Jin X., Yu W. (2018). A multiphase numerical simulation of chloride ions diffusion in concrete using electron microprobe analysis for characterizing properties of ITZ. Constr. Build. Mater..

[20] Xiao J., Li W., Sun Z., Lange D.A., Shah S.P. (2013). Properties of interfacial transition zones in recycled aggregate concrete tested by nanoindentation. Cem. Concr. Compos..

[21] Chung S.-Y., Kim J.-S. (2024). Investigation of gradient properties for cement mortar interfacial transition zones and their effects on mechanical characteristics using micro-CT. Dev. Built Environ..

[22] Wong H.S., Head M.K., Buenfeld N.R. (2006). Pore segmentation of cement-based materials from backscattered electron images. Cem. Concr. Res..

[23] Landis E.N., Nagy E.N., Keane D.T. (2003). Microstructure and fracture in three dimensions. Eng. Fract. Mech..

[24] Brisard S., Serdar M., Monteiro P.J.M. (2020). Multiscale X-ray tomography of cementitious materials: A review. Cem. Concr. Res..

[25] Yue G., Zhang P., Li Q., Li Q. (2018). Performance Analysis of a Recycled Concrete Interfacial Transition Zone in a Rapid Carbonization Environment. Adv. Mater. Sci. Eng..

[26] Zhang H., Zhao Y., Meng T., Shah S.P. (2015). The modification effects of a nano-silica slurry on microstructure, strength, and strain development of recycled aggregate concrete applied in an enlarged structural test. Constr. Build. Mater..

[27] Constantinides G., Ulm F.-J. (2007). The nanogranular nature of C-S-H. J. Mech. Phys. Solids.

[28] Mondal P., Shah S.P., Marks L.D. (2009). Nanomechanical Properties of Interfacial Transition Zone in Concrete. Nanotechnology in Construction 3.

[29] Gan Y., Zhang H., Liang M., Zhang Y., Schlangen E., van Breugel K., Šavija B. (2022). Flexural strength and fatigue properties of interfacial transition zone at the microscale. Cem. Concr. Compos..

[30] Zhang H., Pan Q., Zheng K., Jin C., Pan L. (2022). Mesoscale Study on Splitting Tensile Damage Characteristics of Concrete Based on X-ray Computed Tomography and Digital Image Correlation Technology. Materials.

[31] Ramaniraka M., Rakotonarivo S., Payan C., Garnier V. (2019). Effect of the Interfacial Transition Zone on ultrasonic wave attenuation and velocity in concrete. Cem. Concr. Res..

[32] Ramaniraka M., Rakotonarivo S., Payan C., Garnier V. (2022). Effect of interfacial transition zone on diffuse ultrasound in thermally damaged concrete. Cem. Concr. Res..

[33] Zhang S., Liu R., Lu C., Gao Y., Xu J., Yao L., Chen Y. (2023). Application of digital image correlation to study the influence of the water/cement ratio on the interfacial transition zone in cement-based materials. Constr. Build. Mater..

[34] Shen J., Qin P., Wang Y. (2026). New Insight into the Interfacial Transition Zone in Concrete Based on Fluorescence Microscopy. Appl. Sci..

[35] Jia Z., Han Y., Zhang Y., Qiu C., Hu C., Li Z. (2017). Quantitative characterization and elastic properties of interfacial transition zone around coarse aggregate in concrete. J. Wuhan Univ. Technol.-Mater. Sci. Ed..

[36] Shen Q., Pan G., Zhan H. (2017). Effect of Interfacial Transition Zone on the Carbonation of Cement-Based Materials. J. Mater. Civ. Eng..

[37] Bao H., Xu G., Yu M., Wang Q., Li R., Saafi M., Ye J. (2022). Evolution of ITZ and its effect on the carbonation depth of concrete under supercritical CO_2_ condition. Cem. Concr. Compos..

[38] Momotaz H., Rahman M., Karim M., Zhuge Y., Ma X., Levett P. (2023). Properties of the interfacial transition zone in rubberised concrete—An investigation using nano-indentation and EDS analysis. J. Build. Eng..

[39] Carrara P., De Lorenzis L. (2017). Consistent identification of the interfacial transition zone in simulated cement microstructures. Cem. Concr. Compos..

[40] Bharadwaj A., Sharma M.K., Chakraborty S., Biswas D. (2026). Uncertainty-driven damage estimation of concrete: Integrating realistic aggregate morphology and stochastic flaw behaviour. Int. J. Solids Struct..

[41] Poon C.S., Shui Z.H., Lam L. (2004). Effect of microstructure of ITZ on compressive strength of concrete prepared with recycled aggregates. Constr. Build. Mater..

[42] Sáez del Bosque I.F., Zhu W., Howind T., Matías A., Sánchez de Rojas M.I., Medina C. (2017). Properties of interfacial transition zones (ITZs) in concrete containing recycled mixed aggregate. Cem. Concr. Compos..

[43] Tan X., Hu Z., Li W., Zhou S., Li T. (2020). Micromechanical Numerical Modelling on Compressive Failure of Recycled Concrete Using Discrete Element Method (DEM). Materials.

[44] Giaccio G., Zerbino R. (1998). Failure mechanism of concrete: Combined effects of coarse aggregates and strength level. Adv. Cem. Based Mater..

[45] Wu J., Ding Y., Guo M., Guo S. (2024). Fracture behaviour of modelled interfacial transition zone in recycled aggregate concrete prepared with carbonated aggregate. Constr. Build. Mater..

[46] Xiong X., Xiao Q. (2019). Meso-Scale Simulation of Concrete Based on Fracture and Interaction Behavior. Appl. Sci..

[47] Snyder K.A., Winslow D., Bentz D., Garboczi E. (1992). Effects of Interfacial Zone Percolation on Cement-Based Composite Transport Properties. MRS Online Proc. Libr..

[48] Bentz D.P., Hwang J.T.G., Hagwood C., Garboczi E.J., Snyder K.A., Buenfeld N., Scrivener K.L. (1995). Interfacial Zone Percolation in Concrete: Effects of Interfacial Zone Thickness and Aggregate Shape. MRS Online Proc. Libr..

[49] Garboczi E.J., Bentz D.P. (1997). Analytical formulas for interfacial transition zone properties. Adv. Cem. Based Mater..

[50] Scrivener K.L., Nemati K.M. (1996). The percolation of pore space in the cement paste/aggregate interfacial zone of concrete. Cem. Concr. Res..

[51] Diamond S. (2003). Percolation due to overlapping ITZs in laboratory mortars? A microstructural evaluation. Cem. Concr. Res..

[52] Bentz D.P. (2009). Influence of internal curing using lightweight aggregates on interfacial transition zone percolation and chloride ingress in mortars. Cem. Concr. Compos..

[53] Hillerborg A., Modéer M., Petersson P.-E. (1976). Analysis of crack formation and crack growth in concrete by means of fracture mechanics and finite elements. Cem. Concr. Res..

[54] Bažant Z.P., Oh B.H. (1983). Crack band theory for fracture of concrete. Mater. Struct..

[55] Grassl P., Jirásek M. (2010). Meso-scale approach to modelling the fracture process zone of concrete subjected to uniaxial tension. Int. J. Solids Struct..

[56] Zhang Y., Wang Z., Zhang J., Zhou F., Wang Z., Li Z. (2019). Validation and Investigation on the Mechanical Behavior of Concrete Using a Novel 3D Mesoscale Method. Materials.

[57] Wang J., Jivkov A.P., Engelberg D.L., Li Q. (2022). Image-Based vs. Parametric Modelling of Concrete Meso-Structures. Materials.

[58] Bažant Z.P. (1984). Size effect in blunt fracture: Concrete, rock, metal. J. Eng. Mech..

[59] Grassl P., Grégoire D., Solano L.R., Pijaudier-Cabot G. (2012). Meso-scale modelling of the size effect on the fracture process zone of concrete. Int. J. Solids Struct..

[60] Liu Y., Zhou R., Lu Z., Cheng C., Wang W. (2023). Mesoscale modelling on the evolution of the fracture process zone in concrete using a unified phase-field approach: Size effect study. Theor. Appl. Fract. Mech..

[61] Yu K., Qing L., Hu Y. (2025). The effects of specimen size and aggregate on the evolution of the fracture process zone in concrete: A mesoscale investigation. Compos. Struct..

[62] Schlangen E., van Mier J.G.M. (1992). Simple lattice model for numerical simulation of fracture of concrete materials and structures. Mater. Struct..

[63] Lilliu G., van Mier J.G.M. (2003). 3D lattice type fracture model for concrete. Eng. Fract. Mech..

[64] Leite J.P.B., Slowik V., Mihashi H. (2004). Computer simulation of fracture processes of concrete using mesolevel models of lattice structures. Cem. Concr. Res..

[65] Gupta M., Sharma M., Bishnoi S. (2022). Multiscale modelling of uniaxial compressive stress-strain behaviour of concrete using analytical homogenisation and damage mechanics. Mech. Mater..

[66] Lei J.-J., Wu Z.-X., Wen Z.-J., Cheng Z.-S., Zhu R. (2022). Mesoscale Analysis of the Effect of Interfacial Transition Zone on the Compressive Damage of Concrete Based on Discrete Element Method. Materials.

[67] Liu A., Zou J., Hu W., Liu M., Cong P., Zhang W. (2020). Numerical Simulation of Mesodamage Behavior of Concrete Based on Material Point Method. Adv. Civ. Eng..

[68] Peng Z., Xiong Q.-X., Zhou X., Gao X., Zhao X.-Y., Meng Z., Liu Q.-F. (2025). A porosity-based mechanics model for studying crack evolution from ITZ to mortar matrix in concrete. Comput. Methods Appl. Mech. Eng..

[69] He J., Lei D., Di Luzio G., Zhu F., Bai P. (2022). Mechanical properties measurement and micro-damage characterization of ITZ in concrete by SEM-DIC method. Opt. Lasers Eng..

[70] Wang X., Dong S., Li Z., Han B., Ou J. (2022). Nanomechanical Characteristics of Interfacial Transition Zone in Nano-Engineered Concrete. Engineering.

[71] Zhang H., Gan Y., Xu Y., Zhang S., Schlangen E., Šavija B. (2019). Experimentally informed fracture modelling of interfacial transition zone at micro-scale. Cem. Concr. Compos..

[72] Ren X., Tang C., Xie Y., Long G., Ma G., Wang H., Tang Z. (2024). 3D mesoscale study on the effect of ITZ and aggregate properties on the fracture behaviors of concrete based on discrete element method. J. Build. Eng..

[73] Mishra A., Carrara P., Griffa M., De Lorenzis L. (2026). Fracture in concrete: X-ray tomography with in-situ testing, digital volume correlation and phase-field modeling. Cem. Concr. Res..

[74] Thaue W., Iwanami M., Nakayama K., Yodsudjai W. (2024). Influence of Acetic Acid Treatment on Microstructure of Interfacial Transition Zone and Performance of Recycled Aggregate Concrete. Constr. Build. Mater..

[75] Rangaraju P.R., Olek J., Diamond S. (2010). An investigation into the influence of inter-aggregate spacing and the extent of the ITZ on properties of Portland cement concretes. Cem. Concr. Res..

[76] Sun Y., Qin M., Jia Y., Carlier J.-P., Rougelot T., Shao J.-F. (2026). Coupled hydro-mechanical peridynamic modeling of interfacial transition zone effects on drying shrinkage-induced cracking in concrete. Eng. Fract. Mech..

[77] Kazmi S.M.S., Munir M.J., Wu Y.-F., Patnaikuni I., Zhou Y., Xing F. (2019). Influence of Different Treatment Methods on the Mechanical Behavior of Recycled Aggregate Concrete: A Comparative Study. Cem. Concr. Compos..

[78] Wenzel B., Letelier V., Zambrano G., Bustamante M., Ortega J.M. (2024). Maximizing the Use of Recycled Aggregates from Combined Thermal/Mechanical and Accelerated Carbonation Treatment. Dev. Built Environ..

[79] Alqarni A.S., Abbas H., Al-Shwikh K.M., Al-Salloum Y.A. (2022). Influence of Treatment Methods of Recycled Concrete Aggregate on Behavior of High Strength Concrete. Buildings.

[80] Li X., Zhang Q., Mao S. (2021). Investigation of the bond strength and microstructure of the interfacial transition zone between cement paste and aggregate modified by Bayer red mud. J. Hazard. Mater..

[81] Wang S., Sun Z., Gao Y., Xu X., Yan X., Zhang S. (2024). Effect of Surface Treatment by Mineral Admixture–Magnesium Potassium Phosphate Cement on the Mechanical Properties, Durability, and Microstructure of Recycled Aggregate Concrete. Dev. Built Environ..

[82] Tian F., Chen B. (2025). An FDEM meso-scale fracture mechanism analysis of concrete under compression based on cohesive zone model. Structures.

[83] Burnett T.L., McDonald S.A., Gholinia A., Geurts R., Janus M., Slater T., Haigh S.J., Ornek C., Almuaili F., Engelberg D.L. (2014). Correlative Tomography. Sci. Rep..

[84] Yang Z., Ren W., Sharma R., McDonald S., Mostafavi M., Vertyagina Y., Marrow T. (2017). In-situ X-ray computed tomography characterisation of 3D fracture evolution and image-based numerical homogenisation of concrete. Cem. Concr. Compos..

[85] He J., Lei D., Xu W. (2020). In-situ measurement of nominal compressive elastic modulus of interfacial transition zone in concrete by SEM-DIC coupled method. Cem. Concr. Compos..

